# Determination of sexual dimorphism with CBCT images of the frontal sinus using a predictive formula and an artificial neural network

**DOI:** 10.1590/1678-7757-2025-0049

**Published:** 2025-06-27

**Authors:** Julyana de Araújo OLIVEIRA, Natália Rogério BORELLA, Flávia Maria de Moraes RAMOS-PEREZ, Andrea dos Anjos PONTUAL, Maria Alice Andrade CALAZANS, Felipe Alberto Barbosa Simão FERREIRA, Francisco MADEIRO, Maria Luiza dos Anjos PONTUAL

**Affiliations:** 1 Universidade Federal de Pernambuco Programa de Pós-Graduação em Odontologia Recife PE Brasil Universidade Federal de Pernambuco – UFPE, Programa de Pós-Graduação em Odontologia, Recife, PE, Brasil.; 2 Universidade Federal de Pernambuco Programa de Pós-Graduação em Engenharia Elétrica Recife PE Brasil Universidade Federal de Pernambuco – UFPE, Programa de Pós-Graduação em Engenharia Elétrica, Recife, PE, Brasil.; 3 Universidade Federal Rural de Pernambuco Unidade Acadêmica do Cabo de Santo Agostinho Recife PE Brasil Universidade Federal Rural de Pernambuco – UFRPE, Unidade Acadêmica do Cabo de Santo Agostinho, Recife, PE, Brasil.; 4 Universidade de Pernambuco Escola Politécnica de Pernambuco Recife PE Brasil Universidade de Pernambuco – UPE, Escola Politécnica de Pernambuco, Recife, PE, Brasil.

**Keywords:** Frontal sinus, Machine learning, Forensic anthropology, Neural networks

## Abstract

**Objective:**

this study aims to evaluate the sexual dimorphism of the morphometric features of the frontal sinus via cone beam computed tomography (CBCT) reconstructions, using a predictive formula and an artificial neural network (ANN).

**Methodology:**

the morphometric features of the frontal sinuses obtained from 1,000 CBCT scans, equally distributed by sex, were assessed by two examiners. The frontal sinus morphometric features from 800 CBCT scans were analyzed using Mann-Whitney tests and a multivariate logistic regression model to identify key morphometric features for sex determination and to develop the predictive formula. These features were subsequently used to validate the predictive formula and the machine learning-based classification system. The predictive formula was evaluated using a set of 200 CBCT scans. The machine learning-based classification system consisted of a three-layer ANN trained with 80% of the CBCT scans and tested with the remaining 20%.

**Results:**

Except for the higher frontal sinus index in females, males exhibited higher numerical values for height, width, and anteroposterior (AP) length. The significance level for all statistical tests was set at 0.05. Multivariate logistic regression identified the following four essential morphometric features: sinus height, anteroposterior length (depth) of the sinus, sinus width, and total sinus width. Both the predictive formula and the ANN demonstrated sexual dimorphism. The accuracy, specificity, sensitivity, precision, and F1- score values were 73.50%, 74.00%, 73.00%, 73.74%, and 73.37% for the regression model, and 76.00%, 84.00%, 68.00%, 80.95%, and 73.91% for the ANN, respectively. Except for sensitivity, the ANN outperformed the predictive formula regarding maximum specificity, accuracy, precision, and F1 score.

**Conclusion:**

both methods, particularly the ANN, can potentially support sex estimation in the Brazilian forensic context.

## Introduction

Evaluating sexual dimorphism is crucial for identifying human remains in Forensic Science.^1^ Sex estimation is generally more accurate in adults than in children or adolescents due to hormonal changes during puberty that affect sexual differentiation. This is closely related to the development of skull bones and their anatomical structures, including the facial sinuses.^5,6^

Examining specific human skeletal structures is crucial when only charred human remains are discovered, making fingerprint analysis, pelvic bone studies, or DNA testing impossible.^7^ These structures, such as the frontal sinus, must be unique, unchanging, practical, and definitive, allowing for adequate classification.^10^

Frontal sinuses are anatomical structures that consist of air-filled cavities located between the outer and inner layers of the frontal bone, situated behind the superciliary arches. These sinuses can vary significantly in size and shape from person to person, making them helpful in determining biological sex in humans.^6,11,12^

The development of the frontal sinuses starts in the fourth week of pregnancy and becomes visible on radiographs around the fifth or sixth year of life. Their growth continues until around 20, after which their characteristics remain relatively stable.^2,13,14^ Existing literature indicates that frontal sinuses are generally larger in males. However, caution should be taken when applying specific metrics and morphological features observed in one population to other populations.^15^

Morphometric methods offer greater objectivity than morphological methods, as they rely on consistent measurements that help minimize subjective biases.^3^ Many studies have employed morphometric techniques to determine the sex of individuals across various populations,^2,4,11,16-18^ which demonstrates their superiority over morphological methods.^5,19^

For instance, Tatlisumak, et al.^17^(2007) developed the Frontal Sinus System (FSS), which combines morphological and morphometric analyses of the frontal sinuses. This system evaluates features such as the presence or absence of the frontal sinus (F), the presence of a septum (S), and the number of scallops (S). It also incorporates sinus measurements, including width, height, anteroposterior length, the total width of both sinuses, the distance between the highest points of each sinus, and the distance of each sinus from its maximum lateral limit. Based on morphological and morphometric analyses, this system has demonstrated reproducibility and effectiveness for sex differentiation using radiographs, Multislice Computed Tomography (MCT)^20^and Cone Beam Computed Tomography (CBCT) reconstructions in subsequent studies.^5,10,19,21^

Computed tomography (CT) modalities are recommended for assessing the structure of the paranasal sinuses, especially in advanced research on the frontal sinuses, allowing for multiplanar and three-dimensional evaluations without interference from overlapping structures,^10,11,18,22^ providing higher resolution and enabling precise measurements. CBCT has gained popularity because it exposes patients to a lower radiation dose and offers superior spatial resolution for hard tissues compared to MCT ^10^. Additionally, CBCT machines are user-friendly, have faster scan times, and are more cost-effective, typically ensuring greater patient comfort. Consequently, there has been a notable increase in forensic investigations using CT to determine the sex of unidentified human remains via the morphometric and morphological assessment of the frontal sinuses. In this regard, measurements of the frontal sinuses are considered potentially helpful for sex estimation.^6,12,21,23^ Researchers have encouraged further studies involving diverse populations and representative samples.^20,23,24^

Human factors, such as variations in the perception of gray shades, fatigue, stress, and distraction, can negatively impact the accuracy of imaging assessments.^25^ To address these limitations, computational automation tools, such as artificial neural networks (ANNs), can perform tasks more efficiently than manual methods.^26-28^ ANNs are inspired by the human nervous system. They consist of nodes and connections that learn by adjusting their synaptic weights.^26,27^ Machine learning (ML), a subfield of artificial intelligence, has been applied in various areas, including pattern recognition and clustering. In forensic sciences, ML is utilized to identify an individual’s anthropological characteristics, such as sex and age.^3,4,28,29^

This study aims to evaluate the morphometric features of the frontal sinus to determine sexual dimorphism among Brazilians using CBCT reconstructions. It compares the effectiveness of a predictive formula derived from morphometric evaluations with that of an artificial neural network model. The null hypothesis is that the morphometric features of the frontal sinus do not exhibit significant sexual dimorphism, as assessed by cone beam computed tomography (CBCT) reconstructions using a predictive formula and an artificial neural network (ANN).This research is significant as it employs measurements from CBCT reconstructions of the frontal sinuses to develop and validate a predictive formula and a neural network model for assessing sexual dimorphism.

## Methodology

This study received approval from the Local Research Ethics Committee of the Federal University of Pernambuco in Recife, Pernambuco, Brazil (CAAE number 58438622.4.0000.5208). The research was conducted following the ethical principles outlined in the Declaration of Helsinki by the World Medical Association.

The sample comprised 1,000 CBCT scans, with 500 sourced from female and 500 from male patients. These scans were obtained from a private otorhinolaryngology service database in João Pessoa, Paraíba, Brazil. All CBCT images were captured using the iCAT New Generation^®^ (Imaging Sciences International, Pennsylvania, USA) with the following settings: 120 kV, 5 mA. The images were saved in DICOM (Digital Imaging and Communications in Medicine) format.

CBCT scans had to meet specific criteria to be included in the study sample: (a) the field of view had to encompass the frontal sinus region fully, (b) the voxel size had to be 0.25 mm, and (c) the scans had to be from patients who were at least 20 years old and of Brazilian nationality. Scans exhibiting anomalies in skull development, pathologies, lesions, or evidence of prior surgery in the frontal sinus region were excluded from the study. Additionally, scans with artifacts that complicated or obstructed the evaluation of the sinuses were excluded.

The CBCT scans were prepared to anonymize the patients by removing their sex and age information using OnDemand software (Cybermed, Seoul, Korea). This was accomplished by selecting the “view” option and unchecking “text overlay”. A researcher who was not involved in the CBCT analysis annotated the data. Randomization was conducted using the Randomizer program to ensure a blinded assessment and minimize the risk of bias (Research Randomizer, version 4.0).

### Evaluation of the CBCT images

The frontal sinuses were assessed using the OnDemand software (Cybermed, Seoul, Korea) in a dimly lit, quiet room with a 21.5” LCD monitor with a 1920 x 1080 pixels spatial resolution. The evaluation followed the morphometric analysis proposed by Tatlisumak, et al.^17^(2007). A metric analysis of the frontal sinuses was conducted, measuring the maximum width and height of each of the sinuses (right and left sinus), total width, the distance between the highest points of the sinuses (right and left), and the distance between the highest point and the maximum lateral limit of each sinus. In the axial reconstructions, the anteroposterior length of each sinus was measured. In addition to these variables, the Frontal Sinus Index (FSI) was calculated as the ratio of the maximum height to the maximum width of the frontal sinus.^1,2,4^

The criteria and methodology for evaluating these variables were based on a previous study.^10^ Two experienced and calibrated oral and maxillofacial radiologists assessed the frontal sinuses, with each examiner focusing on one side (either the right or the left). In the intra-examiner analysis, the intraclass correlation coefficient ranged from 0.970 to 1.000, indicating a high level of agreement among the measurements from the same examiner. The inter-examiner correlation coefficient also showed strong agreement, ranging from 0.976 to 1.000. Initially, the thickness for imaging was set at 2.75 mm. Coronal and axial reconstructions were used because they offered the clearest views of the frontal sinuses. Measurements of the maximum width and height of the frontal sinuses were obtained from these reconstructions using the linear measurement tool in the On-Demand software. This method enabled examiners to evaluate the sinuses dynamically by utilizing tools for brightness, contrast, and measurement.

**Figure 1:**
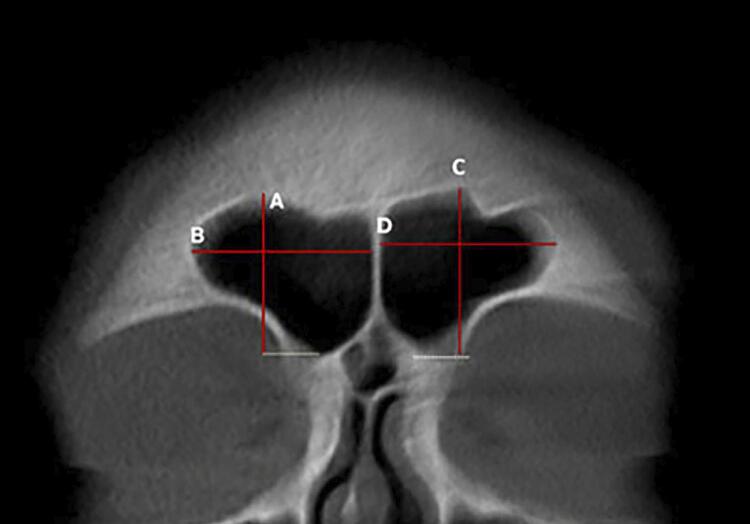
Frontal sinuses visualized in coronal reconstruction. A: Maximum height (MH) of the right sinus; B: Maximum width (MW) of the right sinus; C: Maximum height (MH) of the left sinus; D: Maximum width (MW) of the left sinus.

**Figure 2:**
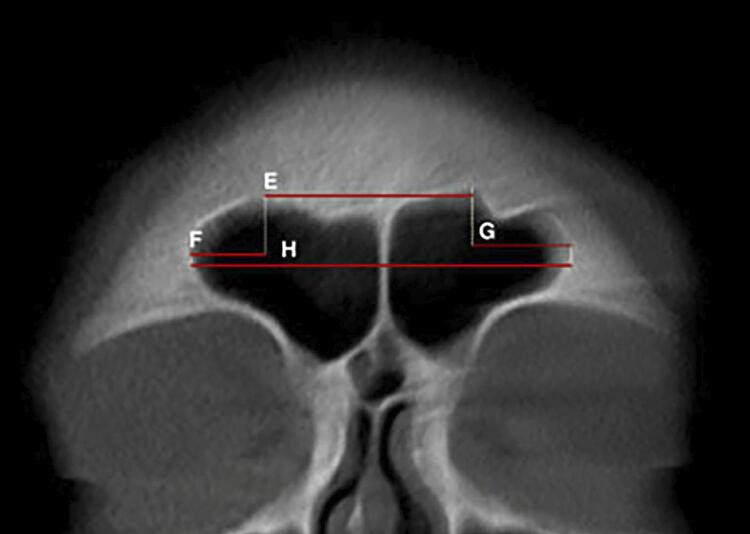
Frontal sinuses visualized in coronal reconstruction. E: Distance between the highest points of the sinuses (Distance 1); F: Distance between the highest point of the right sinus and its maximum lateral limit (Distance 2); G: Distance between the highest point of the left sinus and its maximum lateral limit (Distance 3); H: Total width of the sinuses.

**Figure 3:**
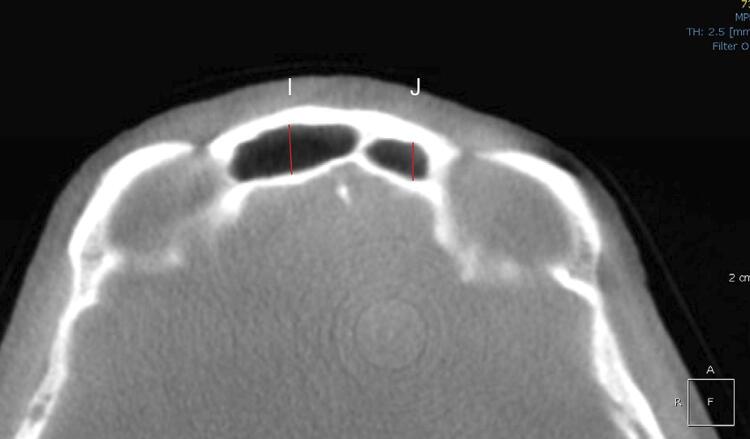
Frontal sinuses visualized in axial reconstruction. I: Anteroposterior (AP) length of the right sinus; J: Anteroposterior (AP) length of the left sinus.

In the coronal reconstructions, the following measurements were taken for each sinus (both right and left): maximum width, maximum height, total width, the distance between the highest points of each sinus, and the distance from the highest point to the maximum lateral limit of each sinus. In the axial reconstructions, the anteroposterior length of each sinus was measured. Additionally, the index from the frontal sinus was calculated as the ratio of the maximum height to the anteroposterior length of the sinus (Figures 1, 2, and 3). All measurements were expressed in millimeters and rounded to two decimal places.

### Data analysis

Data were analyzed using SPSS^®^ (Statistical Package for Social Sciences— International Business Machines, Armonk, New York, USA), version 25, and STATA^®^. The Shapiro-Wilk test was employed to evaluate the normal distribution of data. In contrast, the Chi-square test was used to examine differences in the number of patients across three age groups based on sex. Additionally, the Mann-Whitney test was conducted to analyze the morphometric parameters of the frontal sinus (FS) in relation to sex.

A logistic regression model was then applied, including all variables with a p-value of ≤ 0.20. The model was accepted by the Chi-square test (p<0.001) and was well adjusted according to the Lemeshow test (p=0.790). Four variables were identified as significant during the regression analysis: the average maximum height of the frontal sinuses, the average maximum anteroposterior length of the frontal sinuses, the average maximum width of each sinus, and the total width of the frontal sinus. The regression formula was subsequently used to estimate sexual dimorphism in a test set of 200 samples.

A receiver operating characteristic (ROC) analysis was performed to identify the cutoff point for sexual dimorphism and to calculate the area under the curve (AUC) along with the confusion matrix. The cutoff point value (0.48) was chosen to simultaneously maximize sensitivity and specificity. Metrics such as accuracy, sensitivity, specificity, F1-score, and precision were also calculated. The significance level for all statistical tests was set at 0.05.

### Artificial neural network (ANN) approach

To define the parameters of the proposed artificial neural network (ANN), 21 different configurations were tested to define the number of layers and neurons; four activation functions: identity, logistics, hyperbolic tangent and ReLU; three optimizer options: Limited-memory Broyden-Fletcher-Goldfarb-Shanno (L-BFGS), Stochastic Gradient Descent (SGD) and Adam; and, finally, 10 learning rates. The selected parameters correspond to the scenario with the best combination, consisting of a network with three layers, each with five neurons with the hyperbolic tangent activation function, Adam optimizer, and learning rate of 0.001.

A total of 5,000 was set as the maximum number of epochs. It is known that underfitting may occur if the model is not trained in sufficient time. To avoid the problem of overfitting, an early stop was introduced with a threshold of 10^4^.

Metric data regarding the frontal sinuses and their corresponding sex were compiled to develop the ANN system. The following variables were included:

1. Average maximum height of the frontal sinuses;
2. Average maximum anteroposterior length of the frontal sinuses;
3. Average maximum width of each sinus;
4. Total width of the frontal sinus.

The dataset was divided into two balanced sets according to sex for the simulations: the training set (n=800) was used for the learning process of the model, while the test set (n=200) was utilized to obtain the results. The metrics calculated were consistent with those derived from the regression formula.

## Results

The sample included patients aged between 20 and 101, with a mean age of 47.46, a standard deviation of 16.67, and a median age of 46. The results of the Chi-square test indicated no significant differences in the number of male and female patients across the three age groups (20 to 39, 40 to 59, and 60 years or older) (p=0.377).

Table 1 presents the results of the metric variables analyzed by sex. The Frontal Sinus Index (FSI) had a median value of 3.10 for males, which was lower than the median value of 4.10 for females. Distance 1 did not show a significant difference between the sexes (p=0.052). For the other variables, males exhibited higher values than females in both the right and left frontal sinuses, with p<0.05.

**Table 1:** Distribution of FS morphometric parameters based on sex.

Metric Characteristics of the frontal sinus	Sex						p-Value
	Male (n=400)		Female (n=400)		Total group (n=800)		
	Median	Interquartile range	Median	Interquartile range	Median	Interquartile range	
MH Average	32.51	53.69	28.25	54.36	30.33	57.82	< 0.001*
MW Average	33.08	57.30	27.93	61.35	30.57	63.53	< 0.001*
MAP Length Average	10.31	22.05	6.98	15.63	8.56	22.43	< 0.001*
Distance 1	18.20	45.04	17.20	45.53	17.60	47.17	0.052
Distance 2	21.30	57.29	18.08	52.38	19.56	57.29	< 0.001*
Distance 3	21.25	48.46	17.91	43.80	19.66	50.90	< 0.001*
Total Width	62.17	93.57	52.53	90.08	57.87	99.80	< 0.001*
FSI	3.10	6.75	4.10	8.21	3.55	9.08	< 0.001*

Mann-Whitney Test.

*Significant difference at 5%.

*The variables, except for distance 1, exhibited statistically significant differences between the sexes.

The multivariate logistic regression included the same four key variables as the artificial neural network (ANN). If one sinus was absent, only one sinus measurement was used, and the average was not calculated. Table 2 shows the logistic regression results.

**Table 2:** Results of the logistic regression analysis to determine the sex discrimination function and derive the formula for sex classification.

Fontal sinus Measurements	Coefficient	Standard error	ORa (CI)b	p-Value
Constant	-4.108	0.388	0.016	<0.001*
MH Average	-0.062	0.019	0.940 (0.906-0.975)	0.001*
MAP Length Average	0.542	0.045	1.720 (1.574-1.879)	<0.001*
MW Average	-0.003	0.021	0.997 (0.958-1.038)	0.893
Total Width	0.024	0.011	1.024 (1.002-1.046)	0.031*

Significance at 5%. A: Odds Ration; b Confidence Interval at 95%.

* Variables resulting from logistic regression, along with their respective values, compose the discriminant function for sex.

The equation taken by logistic regression (predicted formula) was as follows:


$$\begin{array}{cc} & \text{ SEX }=\\ & \frac{e^{\left(-4.108-0.062.MH\text{ average }+0.542.MAP\text{ Length average }-0.003.MW\text{ average }+\text{ c. }024\text{ Total Width }\right)}}{1+e^{(-4.108-0.062.\text{ MH aterage }+0.542.\text{ MAP Length average }-0.003.\text{ MW average }-0.024\text{ Total Width }}} \end{array}$$

in which the MH average denotes the average of the maximum height, the MAP length average denotes the average of the maximum anteroposterior length, the MW average denotes the average of the maximum width, and the total width denotes the total width of the frontal sinus.

In binary logistic regression analysis, the constant, which has an estimated value of 4.108, plays a crucial role in modeling the relationship between independent variables and the probability of a binary event occurring. This constant indicates where the logistic function intersects when all independent variables are set to zero, making it essential for minimizing potential biases in the model. Therefore, it is vital to properly weigh the constant in binary logistic regression to ensure accurate and unbiased estimates of the effects of independent variables on the probability of the event.^29,30^

The confusion matrix of the predicted formula was derived using a cutoff point of 0.48, as detailed in Table 3. Besides that, Table 3 also shows the confusion matrix neural network. The predictions exceeding this threshold indicated males (true positives), while those at or below this value indicated females (true negatives). The cell at the intersection of the first row and the first column displays the true-positive cases. A total of 73 individuals were accurately identified as male in the predictive formula while the neural network correctly identified 68 individuals. The next column reflects false negatives: males incorrectly identified as female; the predictive formula presented 27 such cases and the neural network reported 32 cases. The cell corresponding to the second row and column indicates the true negatives. The predictive formula correctly identified 74 women, while the neural network, 84 women. The cell to the left represents false positives; 26 and 16 cases were misclassified as male by predictive formula, and the neural network model, respectively.

**Table 3:** Confusion matrix generated from the predictive formula and neural network model using the test sample.

Methods studied	Real Sex	Sex Predicted		Total
		Male	Female	
Predictive formula	Male	73 (TP)	27 (FN)	100
	Female	26 (FP)	74 (TN)	100
Neural Network	Male	68 (TP)	32 (FN)	100
	Female	16 (FP)	84 (TN)	100

*The male sample was considered positive, and the female sample was negative.

TP=true positive; FN= false negative; FP= False positive; TN=true negative.

**Figure 4:**
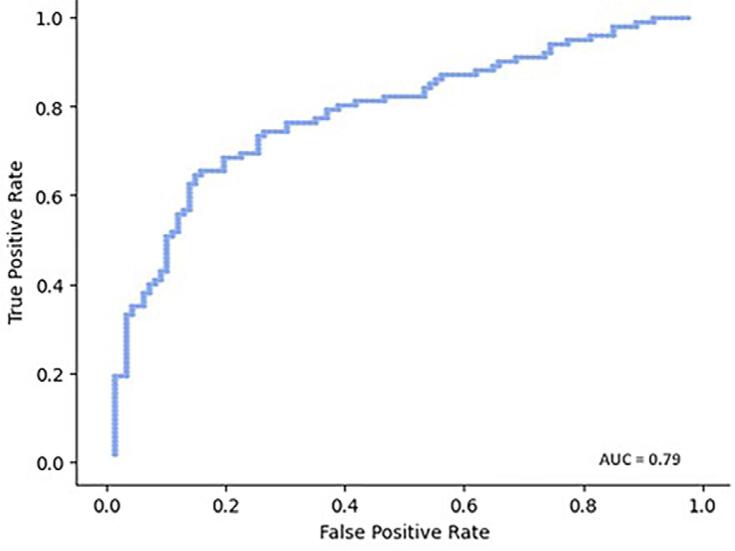
ROC curve of the regression model for frontal sinus in sex estimation using the test sample.

**Figure 5:**
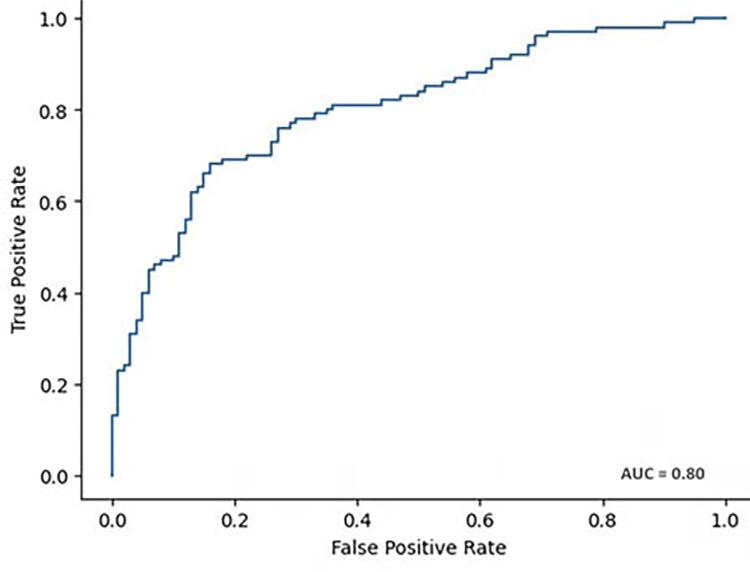
ROC curve of the artificial neural network model for frontal sinus in sex estimation.

The ROC curve of the predicted formula is illustrated in Figure 4, demonstrating an AUC value of 0.79. Regarding the neural network, Figure 5 illustrates the ROC curve based on the true and false positive values, which yields an AUC value of 0.80.

Regarding specificity, the ANN reached 84.00%, while the predictive formula achieved 74.00%. The ANN also demonstrated superiority regarding precision and F1 score.

Moreover, Table 4 provides the performance metrics used to assess the predicted formula and the neural network. The maximum accuracy achieved by the ANN was 76.00%, compared to 73.50% for the regression formula. In terms of specificity, the ANN reached a value of 84.00%, while the regression formula achieved 74.00%. The ANN also demonstrated superiority in terms of precision and F1 score. However, regarding sensitivity, the regression formula performed better, achieving a sensitivity of 73.00%, compared to a maximum of 68.00% for the ANN.

**Table 4:** Metrics for the predictive formula and neural network model obtained from the test sample.

Parameters	Regression model	Neural network	Neural network
**Evaluated**	**(%)**	**Maximum values (%)**	**Average values (%)**
Accuracy	73.50	76.00	72.67
Specificity	74.00	84.00	76.38
Sensitivity	73.00	68.00	68.96
Precision	73.74	80.95	74.67
F1-score	73.37	73.91	71.66

## Discussion

In clinical practice, determining sexual dimorphism is a crucial part of the human identification process. Frontal sinus measurements of a skeleton can add decisive information to this process, especially in cases in which primary identification methods are unusable.^2,3,8^

In this study, both sexual dimorphism methods (predictive formula and ANN) presented good predictive capacity on sexual determination. However, we confirmed the null hypothesis with the majority diagnosis metrics slightly better for the ANN. Only the sensitivity of ANN (68.00%) was lower than the sensitivity of the predictive formula (73.00%). This refers to the lower capability of ANN to identify males compared to the predictive formula. On the other hand, the specificity of ANN is 10% superior to the predictive formula. Consequently, the ANN has more power to detect females than the predictive formula.

Furthermore, we validated both methods. It is essential to conduct validation studies to confirm their performance and to determine whether adjustments are necessary for applying both methods in other populations. To evaluate the applicability of a predictive formula for a different population, such as another country, it is necessary to validate it by evaluating diagnostic metrics, as carried out by Alves, et al.^4^(2023). These authors evaluated the predictive formula developed in a Chinese population^2^ but found low accuracy (53.9%) in a sample Brazilian population. This confirms that specific population characteristics directly influence the applicability of the predictive formula.

CBCT reconstructions enabled the evaluation of the frontal sinuses from multiple planes. Moreover, the morphometrics variables proposed by Tatlisumak, et al.^17^(2007) for assessing the frontal sinuses are applicable and reproducible using this imaging modality. A previous study demonstrated higher reliability and consistency than extraoral techniques.^10^ As a result, a dynamic assessment of the frontal sinuses is feasible and enables precise metric evaluations.^10^

Researchers have successfully utilized measurements of the frontal sinuses (FS) obtained from (CBCT) or (MCT) to assess sexual dimorphism.^5,6,11,15,18,19,21^ However, some studies did not employ sample tests, receiver operating characteristic (ROC) analysis, ROC curve, or other diagnostic metrics to validate their predictive formulas.^5,6,22^ This lack of validation prevents a direct comparison between their results and those of this study.

In this study, the average measurements from the left and right sinuses resulted in a frontal sinus predictive formula that makes its application possible in the case of one, two, or more frontal sinuses. The predictive formula differs from other research presenting right and left frontal sinus measurements.^5,19^ Except for the FSI measurement, all other measurements assessed were significantly higher in males than females. This result agrees with studies conducted in various populations, showing these FS measurements can be a predictive factor.^1,2,4^

In this study, of all measurements studied (MH Average, MW Average, MAP length average, distance 1, distance 2, distance 3, total width and FSI), only MH average, MAP length average, and total width were significant in the logistic regression. This result is similar to the one found in Sri Lankan population.^31^ Furthermore, other studies found additional significant measurements in the predictive formula. Besides these measurements, the distance between the highest point of the right sinus and its maximum lateral limit exhibited sexual dimorphism in the Indian population.^19^ In addition, the Iraqui^18^ and Egyptian^5^ populations presented almost all measurements initially evaluated in the present study.^18^

Our study showed dimorphic potential for anteroposterior length (AP), which aligns with research conducted in the Indian,^19^ Nigerian,^32^ and Egyptian^6^ populations. FSI, on the other hand, was characterized as the only metric variable that was higher in females. This fact can be explained by the higher anteroposterior length in males, which is inversely proportional to the FSI. The proportionality involving the height and anteroposterior length measurements in males may further explain these findings.

The AUC of 0.79 was closer to the value observed in 130 CBCT scans from southeastern Brazil,^21^ which reported an AUC of 0.80, with the total volume being the predominant variable. In contrast, in the Egyptian sample of 100 CBCT scans, the predictive formula based solely on frontal sinus (FS) height yielded an AUC of 0.50.^15^ In this study, the predictive formula achieved an accuracy of 73.50%. This accuracy was higher than that reported in a study involving 140 MCT scans from northeast Brazil, where the regression formula using FS height and AP length had accuracies of 61.00% and 58.00%, respectively^20^. Additionally, when analyzing cephalometric radiographs from southeastern Brazilians, the predictive formula yielded an accuracy of 70.20%.^4^

Systems based on ANN have been presented as an essential engineering and computing tool in Forensic Anthropology. They assist professionals in forensic dentistry by improving their work processes.^33^ In the literature, only one study involved using ANN and frontal sinus measurements to determine sexual dimorphism^4^. However, the previous study used 255 cephalometric radiographs from southeast Brazilians and obtained an accuracy of 73.30%, which is lower than that achieved in this study (76.00%). This difference in the results can be attributed to the types of exams and sample size used in this study.

To the best of our knowledge, only one study has utilized convolutional neural networks to determine sexual dimorphism based exclusively on data obtained from frontal sinus computed tomography (CT) scans.^38^ Several key characteristics distinguish our work from this study mentioned in the literature. The previous study had a relatively small sample size, comprising 310 CBCT scans from South Korean patients, and did not compare its findings with conventional methods, such as the predictive formula. In contrast, this study evaluates the neural network approach using a predictive formula, with measurements taken by examiners from an extensive database of 1,000 CT scans.

There are a few limitations that should be considered. Firstly, the sample was exclusively composed of Brazilian individuals, which may limit the application of findings to other populations with different features. Furthermore, while CBCT is recognized for its high spatial resolution and affordability, the technique is still liable to artifacts and fluctuations in image quality, which can affect measurement accuracy. Besides, relying on radiologists to evaluate the CBCT scans introduces subjectivity, even though examiner agreement is generally high.

Although this study provides valuable insights into sexual dimorphism via CBCT scans, possible limitations should be considered. We did not assess the FS morphological parameters, which may have influenced the results. However, some authors did not find the significance of the morphological parameters between the sexes, or it did not remain in the logistic regression with the morphometric parameters.^5,19^

Furthermore, the decision to exclude these parameters was based on the variability observed among examiners when evaluating imaging tasks, as their experience level can significantly impact performance. Additionally, evaluating multiple variables can be time-consuming. Therefore, only the metric variables proposed by Tatlisumak, et al. (2007)^1^⁷ were selected to minimize subjectivity in image evaluation and streamline the assessment process. In this study, inter-examiner agreement regarding the analysis of the metric parameters was found to be nearly at the maximum desirable level.

Using CNNs increases computational complexity, requiring greater execution time and cost. In addition, this type of network is often used for problems in which images are the network input.^38,39^ Regarding the model produced, tabular data presented a solution with low computational complexity. However, the possibility of using CNN with images as input could be promising for future research.

Although the artificial neural network (ANN) has surpassed the predictive formula in specific metrics, its practical application in the routine of dentists would be more accessible if an interface were developed to facilitate usability for health professionals. The development and use of software for the automatic classification of exams to support decision-making is relevant, as it is a quick and low-cost solution. Additionally, it minimizes human subjectivity during analysis.

On the other hand, this study presents a significant and well-balanced dataset, ensuring robust statistical analysis. It also compares two methods—a predictive formula and an artificial neural network (ANN)—for determining sexual dimorphism via the morphometry of the frontal sinus using CBCT scans. The ANN demonstrates sufficient specificity and precision in this context. The study could be expanded to include a broader range of populations and incorporate additional anatomical variables, such as other paranasal sinuses, to further refine the accuracy of the model. Furthermore, artificial intelligence has the potential to improve both the speed and accessibility of forensic investigations.

## Conclusion

This study has shown encouraging results in the use of the frontal sinus to determine sexual dimorphism. In the two proposed methods, it was possible to distinguish sex based on frontal sinus measurements. Except for IEF, the morphometric measurements evaluated were higher in males. The average maximum height, the anteroposterior length, and the total width of the frontal sinus serve as indicators of sexual dimorphism.

Regarding the methods studied, the predictive formula and the artificial neural network (ANN) proved effective in determining sexual dimorphism. Except for sensitivity, the ANN performs slightly better than the predictive formula in terms of maximum specificity, accuracy, precision, and F1 score. The two methods proposed, especially ANN, can help estimate sex in Brazilian forensic investigations.

In relation to qualitative and quantitative morphological variables, we have started two complementary studies with different objectives: one focusing exclusively on morphometric analysis and the other on morphological assessment, incorporating qualitative and quantitative variables. These ongoing investigations aim to improve the accuracy of sex prediction using a predictive formula and an artificial neural network (ANN) model.

However, it is important to note that the sample consisted exclusively of individuals from the Northeast region of Brazil, which is insufficient to guarantee the generalizability of the method to other populations and ethnic groups. Therefore, previous validation studies involving more diverse and representative populations are essential before the predictive formula and ANN model can be reliably applied in wider forensic contexts.

## References

[1] Kiran CS, Ramaswamy P, Khaitan T (2014). Frontal sinus index: a new tool for sex determination. J Forensic Radiol Imaging.

[2] Luo H, Wang J, Zhang S, Mi C (2018). The application of frontal sinus index and frontal sinus area in sex estimation based on lateral cephalograms among Han nationality adults in Xinjiang. J Forensic Leg Med.

[3] Toneva DH, Nikolova SY, Agre GP, Zlatareva DK, Hadjidekov VG, Lazarov NE (2020). Data mining for sex estimation based on cranial measurements. Forensic Sci Int.

[4] Alves CP, Costa C, Michel-Crosato E, Biazevic MG (2023). Use of the frontal sinus to evaluate sexual dimorphism in a Brazilian sample. Forensic Imaging.

[5] Motawei SM, Wahba BA, Aboelmaaty WM, Tolba EM (2016). Assessment of frontal sinus dimensions using CBCT to determine sexual dimorphism amongst Egyptian population. J Forensic Radiol Imaging.

[6] Ibrahim MA, Abdel-Karim RI, Ibrahim MS, Dar UF (2020). Comparative study of the reliability of frontal and maxillary sinuses in sex identification using multidetector computed tomography among Egyptians. Forensic Imaging.

[7] Spradley MK, Jantz RL (2011). Sex estimation in forensic anthropology: skull versus postcranial elements. J Forensic Sci.

[8] Prajapati G, Sarode SC, Sarode GS, Shelke P, Awan KH, Patil S (2018). Role of forensic odontology in the identification of victims of major mass disasters across the world: a systematic review. PLoS One.

[9] Heathfield LJ, Haikney TE, Mole CG, Finaughty C, Zachou AM, Gibbon VE (2021). Forensic human identification: investigation into tooth morphotype and DNA extraction methods from teeth. Sci Justice.

[10] Soares CB, Almeida MS, Lopes Pde M, Beltrão RV, Pontual Ados A, Ramos-Perez FM (2016). Human identification study by means of frontal sinus imaginological aspects. Forensic Sci Int.

[11] Tatlisumak E, Asirdizer M, Bora A, Hekimoglu Y, Etli Y, Gumus O (2017). The effects of gender and age on forensic personal identification from frontal sinus in a Turkish population. Saudi Med J.

[12] Prado PS, Adams K, Fernandes LC, Kranioti E (2021). Frontal sinus as an identity and sex indicator. Morphologie.

[13] Silva RF, Prado FB, Caputo IG, Devito KL, Botelho TL, Daruge E (2009). The forensic importance of frontal sinus radiographs. J Forensic Leg Med.

[14] Buyuk SK, Karaman A, Yasa Y (2017). Association between frontal sinus morphology and craniofacial parameters: a forensic view. J Forensic Leg Med.

[15] ElBeshlawy DM, Helaly YR (2020). Frontal sinus index for sex estimation: Is it possible?. Forensic Imaging.

[16] Emekli E (2023). Sex determination using frontal sinus diameters on direct radiography. Cureus.

[17] Tatlisumak E, Yilmaz Ovali G, Aslan A, Asirdizer M, Zeyfeoglu Y, Tarhan S (2007). Identification of unknown bodies by using CT images of frontal sinus. Forensic Sci Int.

[18] Uthman AT, Al-Rawi NH, Al-Naaimi AS, Tawfeeq AS, Suhail EH (2010). Evaluation of frontal sinus and skull measurements using spiral CT scanning: an aid in unknown person identification. Forensic Sci Int.

[19] Hemanthakumar S, Gopal K, Kumar P (2022). Assessment of sexual dimorphism using 3D CBCT image data among Indians. Bioinformation.

[20] Mendonça DS, Kurita LM, Carvalho FS, Tuji FM, Silva PG, Bezerra TP (2021). Development and validation of a new formula for sex estimation based on multislice computed tomographic measurements of maxillary and frontal sinuses among Brazilian adults. Dentomaxillofac Radiol.

[21] Choi IG, Duailibi-Neto EF, Beaini TL, Silva RL, Chilvarquer I (2018). The frontal sinus cavity exhibits sexual dimorphism in 3D Cone-beam CT images and can be used for sex determination. J Forensic Sci.

[22] Singh PK, Paudel RC, Menezes RG (2021). Predictability of sex from frontal sinus in nepalese population. Kathmandu Univ Med J (KUMJ).

[23] Mendonça DS, Ribeiro EC, Barros Silva PG, Rodrigues AA, Kurita LM, Aguiar AS (2022). Diagnostic accuracy of paranasal sinus measurements on multislice computed tomography for sex estimation: a systematic review, meta-analysis, and meta-regression. J Forensic Sci.

[24] Sridhar M, Bagewadi A, Lagali-Jirge V, Kumar SL, Panwar A, Keluskar V (2023). Reliability of gender determination from paranasal sinuses and its application in forensic identification-a systematic review and meta-analysis. Forensic Sci Med Pathol.

[25] Birdal RG, Gumus E, Sertbas A, Birdal IS (2016). Automated lesion detection in panoramic dental radiographs. Oral Radiol.

[26] Mohammad N, Ahmad R, Kurniawan A, Mohd Yusof MY (2022). Applications of contemporary artificial intelligence technology in forensic odontology as primary forensic identifier: a scoping review. Front Artif Intell.

[27] Haykin S (2008). Neural networks and learning machines.

[28] Silva G, Oliveira L, Pithon M (2018). Automatic segmenting teeth in X-ray images: Trends, a novel data set, benchmarking and future perspectives. Expert Syst Appl.

[29] Kim S, Lee YH, Noh YK, Park FC, Auh QS (2021). Age-group determination of living individuals using first molar images based on artificial intelligence. Sci Rep.

[30] Srimaneekarn N, Hayter A, Liu W, Tantipoj C (2022). Binary response analysis using logistic regression in dentistry. Int J Dent.

[31] Wickramasinghe C, Vadysinghe AN, Kodikara S, Udupihilla J (2022). Frontal sinus pattern analysis for human identification using non-contrast computed tomography images: A Sri Lankan experience. SAGE Open Med.

[32] Anyanwu GE, Ezeofor SN, Obikili EN, Ugbor EV (2021). Morphometric Evaluation of frontal and maxillary sinuses and bizygomatic distance of igbos in south-east and ogonis in south-south Nigeria using computerized tomography scan. J Adv Med Med Res.

[33] Khanagar SB, Vishwanathaiah S, Naik S, Al-Kheraif AA, Divakar DD, Sarode SC (2021). Application and performance of artificial intelligence technology in forensic odontology: a systematic review. Leg Med (Tokyo).

[34] Galante N, Cotroneo R, Furci D, Lodetti G, Casali MB (2023). Applications of artificial intelligence in forensic sciences: current potential benefits, limitations and perspectives. Int J Legal Med.

[35] Ketsekioulafis I, Filandrianos G, Katsos K, Thomas K, Spiliopoulou C, Stamou G (2024). Artificial Intelligence in Forensic Sciences: a systematic review of past and current applications and future perspectives. Cureus.

[36] Tynan P (2024). The integration and implications of artificial intelligence in forensic science. Forensic Sci Med Pathol.

[37] Singh S, Singha B, Kumar S (2024). Artificial intelligence in age and sex determination using maxillofacial radiographs: a systematic review. J Forensic Odontostomatol.

[38] Silva RL, Yang S, Kim D, Kim JH, Lim SH, Han J (2024). Automatic segmentation and classification of frontal sinuses for sex determination from CBCT scans using a two-stage anatomy-guided attention network. Sci Rep.

[39] Hamidi O, Afrasiabi M, Namaki M (2024). GADNN: a revolutionary hybrid deep learning neural network for age and sex determination utilizing cone beam computed tomography images of maxillary and frontal sinuses. BMC Med Res Methodol.

